# Case of Traumatic Abscess of the Liver, with Thoraco Abdominal Fistula

**Published:** 1860-01

**Authors:** H. Wardner

**Affiliations:** Demonstrator of Anatomy in Lind University, Chicago


### A CASE OF TRAUMATIC ABSCESS OF THE LIVER, WITH THORACICO-ABDOMINAL FISTULA.

BY H. WARDNER, M. D.,
Demonstrator of Anatomy in Lind University, Chicago.

On the 13th of October, 1857, I was called at 2 o'clock, A. M. to see a man who had been wounded by a pistol containing two balls, about five hours previous. Found that one ball had entered over the ninth rib, just escaping its angle, and glancing from the bone could be traced about four or five inches under the integument, in the direction of the right nipple. The other ball had entered the body between the ninth and tenth ribs, about three inches from their angles. The hole made by this ball could not be traced more than an inch or so, and appeared to pass into the trunk. On the third day, I removed one ball, which I judged to be the first one mentioned, from the lower edge of the seventh rib, at the junction with its cartilage. The other has never been found.

When first visited, the patient was in much pain, with short and hurried respiration: but he apparently improved up to the time of removing the ball, at which time a cough was observed to trouble him, and the day following was accompanied by an expectoration of thick yellowish matter, occasionally streaked with blood. The treatment up to this time had been anodyne and antiphlogistic. The wounds kept open by a tent.

On the fifth day, a chill occurred, and returned for three successive days. Gave quinine in full doses.

Oct. 21st, eighth day. He had two chills, and for three days following the chills irregular and frequent, attended with watchfulness and restlessness. I concluded that these chills were in consequence of the formation of *pus* in the system, and consequently the patient was put upon tonics, with ale and wine and a generally nourishing diet, with expectorants and anodynes as circumstances required.

On the thirteenth day I learned that the patient had been very restless during the preceding night, and vomited considerable. The attendant stated, that during the vomiting wind passed from the lower or second ball hole, with nearly sufficient force to blow out a candle. There was some feverish excitement at the time I saw him.

On the fourteenth day I found a free discharge of pus from the lower hole. On the day following the discharge had much increased. There was much restlessness. Gave anodynes and arterial sedatives. On the sixteenth day, bile was discharging with the pus.

On the seventeenth day, the patient was dull and feverish, the discharge had assumed a muco-bilious appearance, about the consistence of the white of the egg; there was occasionally streaks of mucous or albuminous discharge not stained with bile. The symptoms at this time were more unfavorable, bowels badly constipated, urine scanty and high colored, cough quite severe, with copious mucous expectoration; skin dry and feverish; intellect dull and occasionally wandering. These symptoms, however, all passed off after the action of suitable medicines. The discharge of bile continued for two weeks, gradually diminishing, and there was a watery serous discharge for ten days longer. The hole was then filled with "*proud flesh*," which, every day or two, would protrude an inch or more. This was removed by the knife as often as every two days, and the orifice cauterized with nitrate of silver.

Dec. 4th. "The wound has healed, and the patient is able to be up and walk about. The feet and legs were swollen at times, and the face has a bloated appearance. There is some cough, and a little expectoration of mucous." He was directed to use ale daily, also the calybeates, and iod. potassa, and to exercise as much as his strength would permit.

I saw the patient on the 16th of February, following. The dropsical symptoms had gradually disappeared; the face, however, was a little fuller than natural; eye dull and languid; said his appetite was good and bowels regular, but that he was easily wearied by any exertion.

This man has been able to work for a year and a half, and when last seen was in good health, and in the act of leaving the city, "a la Francais," without paying his doctor.

In considering this case, it is evident that the ball must have penetrated the right pleural cavity, passed through lung, and also through the diaphragm into the liver; thus opening communication between the thoracic and abdominal cavities. The time from the injury to the formation of the abscess was sufficient for adhesive inflammation to unite the opposing peritonial and pleural surfaces, and a channel for the exit of pus through the wound was thus formed, so that there was no escape of it into either cavity. To this circumstance the man undoubtedly owes his life.

A wounded portion of the lung must have opened into the channel, giving passage to the air that was expelled during the emesis.

The biliary ducts must have been injured to a considerable extent. The subsequent dropsical symptoms led us to infer that the circulation of the blood through the organ was partly destroyed, either by the wound, or cicatrization, or both; and the final disappearance of these symptoms must have been owing to the re-establishment of the circulation sufficiently to allow the organ to carry on its functions in nearly a normal condition.

The man was troubled with a cough for nearly a year, particularly when exercising; this I attributed to the fact that as the liver, lower portion of the right lung, and diaphragm, were firmly attached to the thoracic walls by the cicatrized adhesions, motion of the body must necessarily produce irritation of those organs, until they become accustomed to their new attachments. The case I consider unique, and of interest in showing the *vis mendicatrix naturæ.*

